# Genomic landscape of the signals of positive natural selection in populations of Northern Eurasia: A view from Northern Russia

**DOI:** 10.1371/journal.pone.0228778

**Published:** 2020-02-05

**Authors:** Andrey V. Khrunin, Gennady V. Khvorykh, Alexei N. Fedorov, Svetlana A. Limborska

**Affiliations:** 1 Department of Molecular Bases of Human Genetics, Institute of Molecular Genetics of Russian Academy of Sciences, Moscow, Russia; 2 Department of Medicine, University of Toledo, Toledo, Ohio, United States of America; Universitat Pompeu Fabra, SPAIN

## Abstract

Natural selection of beneficial genetic variants played a critical role in human adaptation to a wide range of environmental conditions. Northern Eurasia, despite its severe climate, is home to lots of ethnically diverse populations. The genetic variants associated with the survival of these populations have hardly been analyzed. We searched for the genomic signatures of positive selection in (1) the genome-wide microarray data of 432 people from eight different northern Russian populations and (2) the whole-genome sequences of 250 people from Northern Eurasia from a public repository through testing the extended haplotype homozigosity (EHH) and direct comparison of allele frequency, respectively. The 20 loci with the strongest selection signals were characterized in detail. Among the top EHH hits were the *NRG3* and *NBEA* genes, which are involved in the development and functioning of the neural system, the *PTPRM* gene, which mediates cell–cell interactions and adhesion, and a region on chromosome 4 (chr4:28.7–28.9 Mb) that contained several loci affiliated with different classes of non-coding RNAs (*RN7SL101P*, *MIR4275*, *MESTP3*, and *LINC02364*). *NBEA* and the region on chromosome 4 were novel selection targets that were identified for the first time in Western Siberian populations. Cross-population comparisons of EHH profiles suggested a particular role for the chr4:28.7–28.9 Mb region in the local adaptation of Western Siberians. The strongest selection signal identified in Siberian sequenced genomes was formed by six SNPs on chromosome 11 (chr11:124.9–125.2 Mb). This region included well-known genes *SLC37A2* and *PKNOX2*. *SLC37A2* is most-highly expressed in the gut. Its expression is regulated by vitamin D, which is often deficient in northern regions. The *PKNOX2* gene is a transcription factor of the homeobox family that is expressed in the brain and many other tissues. This gene is associated with alcohol addiction, which is widespread in many Northern Eurasian populations.

## Introduction

In the last decade, human genetic diversity has been extensively studied at both the global and regional level using genome-wide panels of single-nucleotide polymorphisms (SNPs) and whole-genome sequences. A large amount of information has been obtained on the genetic structure of different populations and several models of their relationships have been proposed, including possible routes and waves of human migrations [1–12]. In addition to population demography, natural selection is an important force that shapes the genetic variability of populations. It leads to changes in the allele frequencies of particular loci if they are adaptive and increase population fitness in specific environmental conditions [13]. The identification of genomic loci that are subjected to selection provides deeper insights into the existence of certain population genetic structures [14]. These loci can be also instrumental for understanding the genetic bases of population-level differences in the distribution of common diseases and traits [15–19]. Therefore, establishing a broad picture of local human genetic adaptations is a very important objective. To date, there are few local adaptations whose phenotypes or types of selective pressure are characterized well and are referred to the corresponding genomic context [20]. However, many genomic regions that have been described as being subjected to selection (e.g., containing certain molecular types of selection signatures) remain poorly understood, including loci that have been associated with specific common diseases and traits [21]. Moreover, many additional genomic regions are expected to be identified as being targets of selection, particularly during the exploration of the patterns of genetic variation in populations that have yet to be sampled.

**Fig 1:**
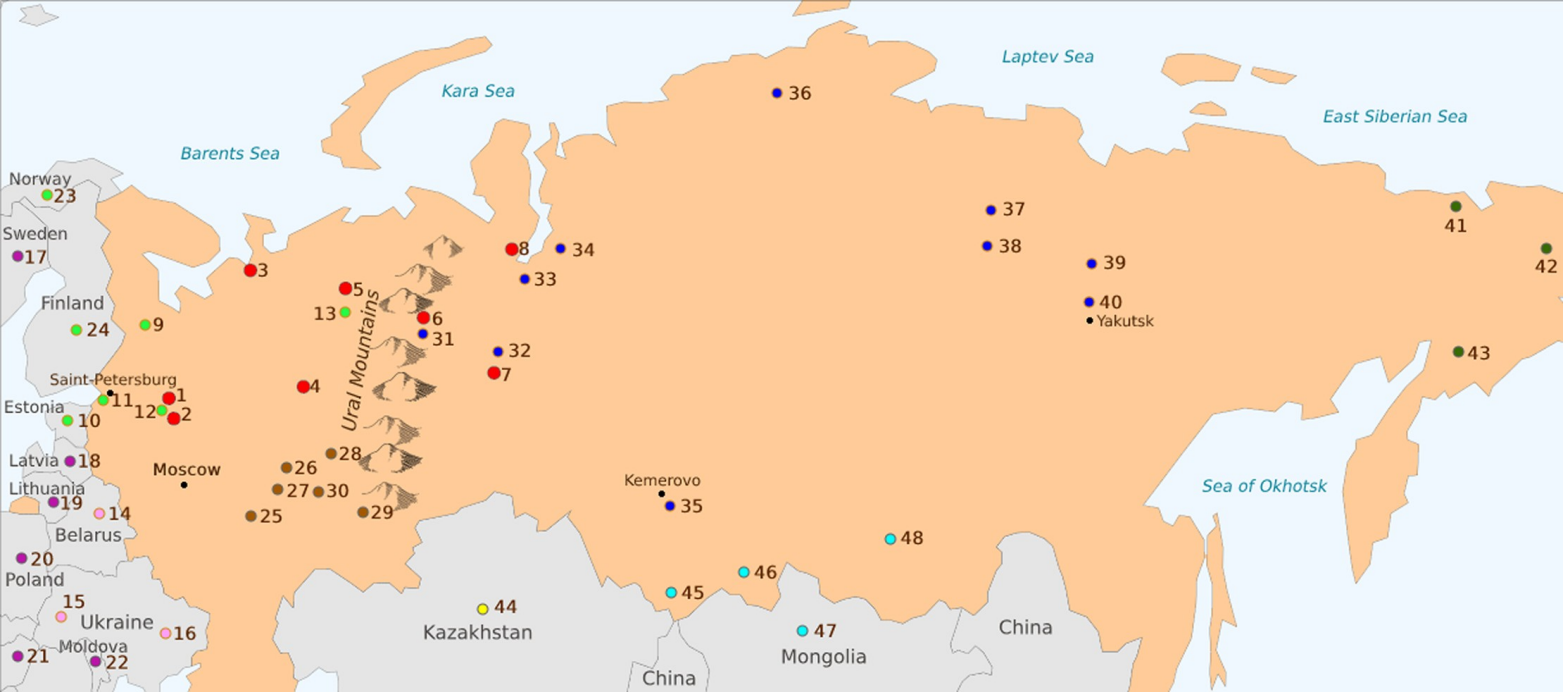
Geographical locations of the populations and samples studied. The numbers denote the following population samples: 1, Veps; 2, Russians from Ustyuzhna, Vologda region; 3, Russians from Mezen, Archangelsk region; 4, Priluzski Komi from Ob’yachevo District, Komi Republic; 5, Izhemski Komi from Izhma district, Komi Republic; 6, Mansi from Khanty-Mansi Autonomous Okrug; 7, Khanty from Khanty-Mansi Autonomous Okrug; 8, Nenets from Yamalo-Nenets Autonomous Okrug; 9, Karelians; 10, Estonians; 11, Ingrians; 12, Vepsas; 13, Komis; 14, Belarusians; 15, Ukrainians West; 16, Ukrainians East; 17, Swedes; 18, Latvians; 19, Lithuanians; 20, Poles; 21, Hungarians; 22, Moldavians; 23, Saami; 24, Finnish; 25, Mordvins; 26, Maris; 27, Chuvashes; 28, Udmurds; 29, Bashkirs; 30, Tatars; 31, Mansis; 32, Khantys; 33, Selkups; 34, Nenets; 35, Shor; 36, Nganasans; 37, Evenks; 38, Evens; 39, Sakha; 40, Kets; 41, Eskimo; 42, Chukchis; 43, Koryaks; 44, Kazakhs; 45, Altaians; 46, Tuvinians; 47, Mongolians; 48, Buryats. The numbers from 1 to 8 represent the samples genotyped by authors with microarrays and the rest were obtained from the Estonian Biocentre Human Genome Diversity Panel (EGDP [5,29]). The colors represent the following populations and groups of samples: red–datasets obtained with microarrays, sky blue–Altaians, yellow–Asia, deep green–Chukchi, magenta–Europe, light green–samples from North East Europe populations (NEE group), blue–samples from North West Asia populations (NWA group), pink–Slavs, brown–Tatar. The complete list of samples taken from the EGDP is presented in S3 Table (i.e., samples of non-Eurasian geography). The map was generated using R package tmap (v2.3–1) [30].

From this point of view, Russia is one of the most promising geographic areas. Its vast territory is peopled by 195 ethnically diverse indigenous human populations [22] living in very heterogeneous climatic conditions. Moreover, this area was the arena for ancient gene flow events directed not only toward Europe, but also toward the Americas, where they became the blueprint of Native American ancestry [8,10,23]. Until recently, Russia remained a white spot on the genomic map of human local adaptations. Although this situation has started to change, most studies have focused on the examination of the genetic diversity of Eastern Siberian indigenous populations. These studies searched for the footprints of selection that allowed these peoples to survive in an extremely cold environment [24–26]. In this article, we present the results of the scanning of the genome-wide microarray data of eight populations from the Northern European part of Russia and from Western Siberia (Subarctic Uralic region) for regions with signatures of natural selection (Fig 1). Local adaptation in those two regions of Russia has never been studied at this level. Western Siberian populations—Khanty, Mansi, and Nenets—were of particular interest, as they were recently shown to occupy a distinct position among Northern Eurasian populations because they exhibited the greatest percentage of ancestry attributable to Ancient Northern Eurasians [10]. We identified the genomic loci under selection pressure using two tests that contrasted the profiles of the extended haplotype homozygosity (EHH) of the ancestral and derived alleles at the SNP of interest in a single population (iHS test) or between two populations (XP-EHH test) [27,28].

The densest modern SNP microarrays (e.g., Illumina Infinium Omni5 microarrays; Illumina Inc., USA) allow the processing of up to 4.3 million polymorphic sites. However, they represent about 1% of all known human SNPs [31]. Using this approach, researchers mainly register not the SNPs that undergo selection, but the neighboring SNPs that are presented on the chip and are in linkage disequilibrium with the SNPs in question, which are the original targets of selection. This drawback may be overcome by using whole-genome sequencing data. However, this method is considerably more expensive, hence, for studying hundred(s) of individuals it may not be feasible. Here, we took advantage of the public data from the Estonian Biocentre Human Genome Diversity Panel (EGDP), which contains multiple completely sequenced genomes from Russia and neighboring populations [5,29]. In this database, loci under selection were searched based on the differences in their allele frequencies between populations. The combination and comparisons of both technologies allowed us to obtain good insight into the selection genetics in Northern Eurasia, particularly in the Russian Northern territories.

## Results

### Genomic regions where signals of selection were revealed using the iHS test

**Fig 2:**
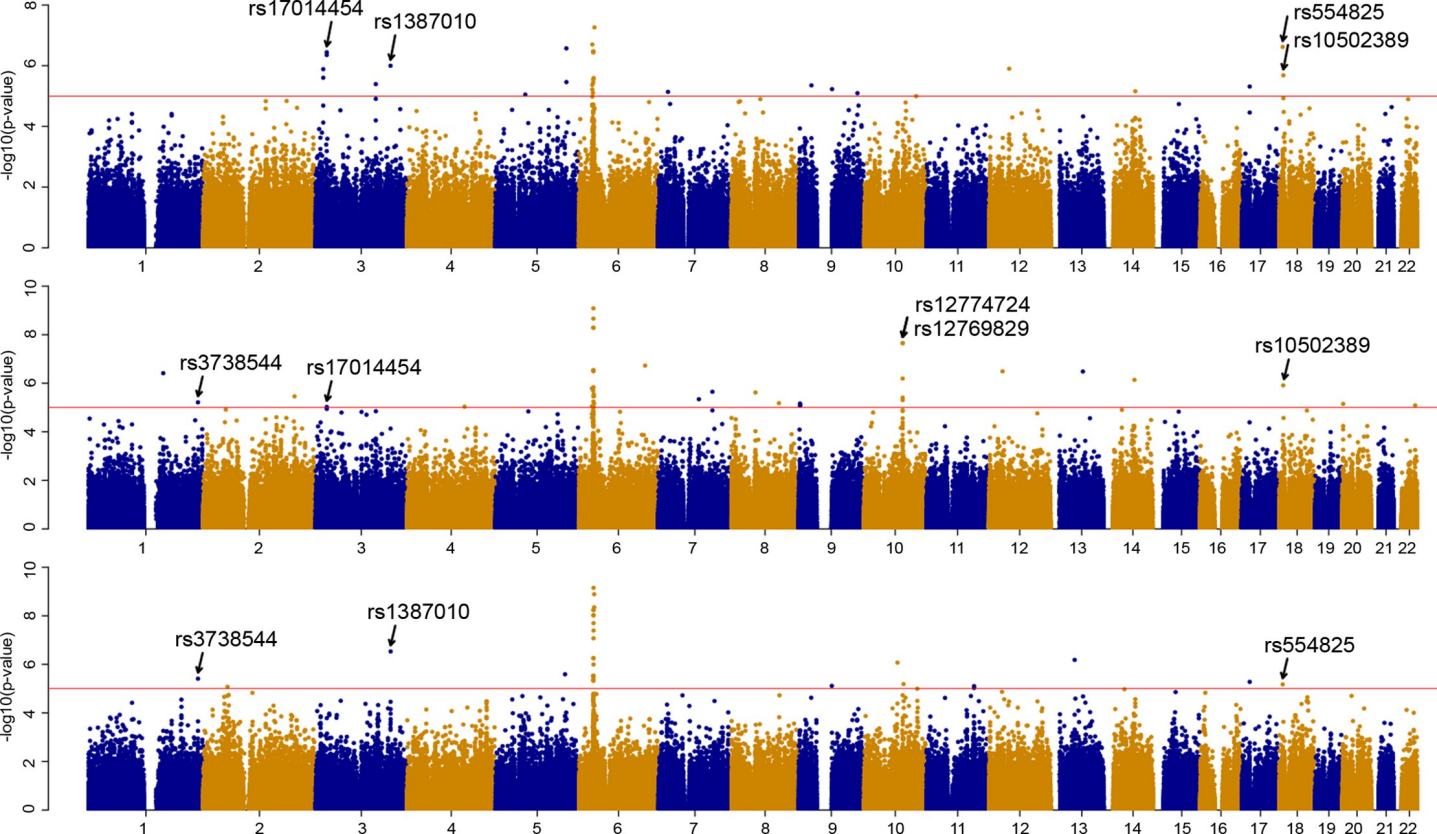
Genome-wide (autosomes 1–22) distribution of p-values for iHS scores in three out of the eight populations studied. The populations are (from top to bottom): Russians from Mezen, Russians from Ustyuzhna, and Veps. Horizontal red lines indicate P-value threshold applied (*P* ≤ 1 × 10^−5^). Loci of interest are pointed with arrows.

**Fig 3:**
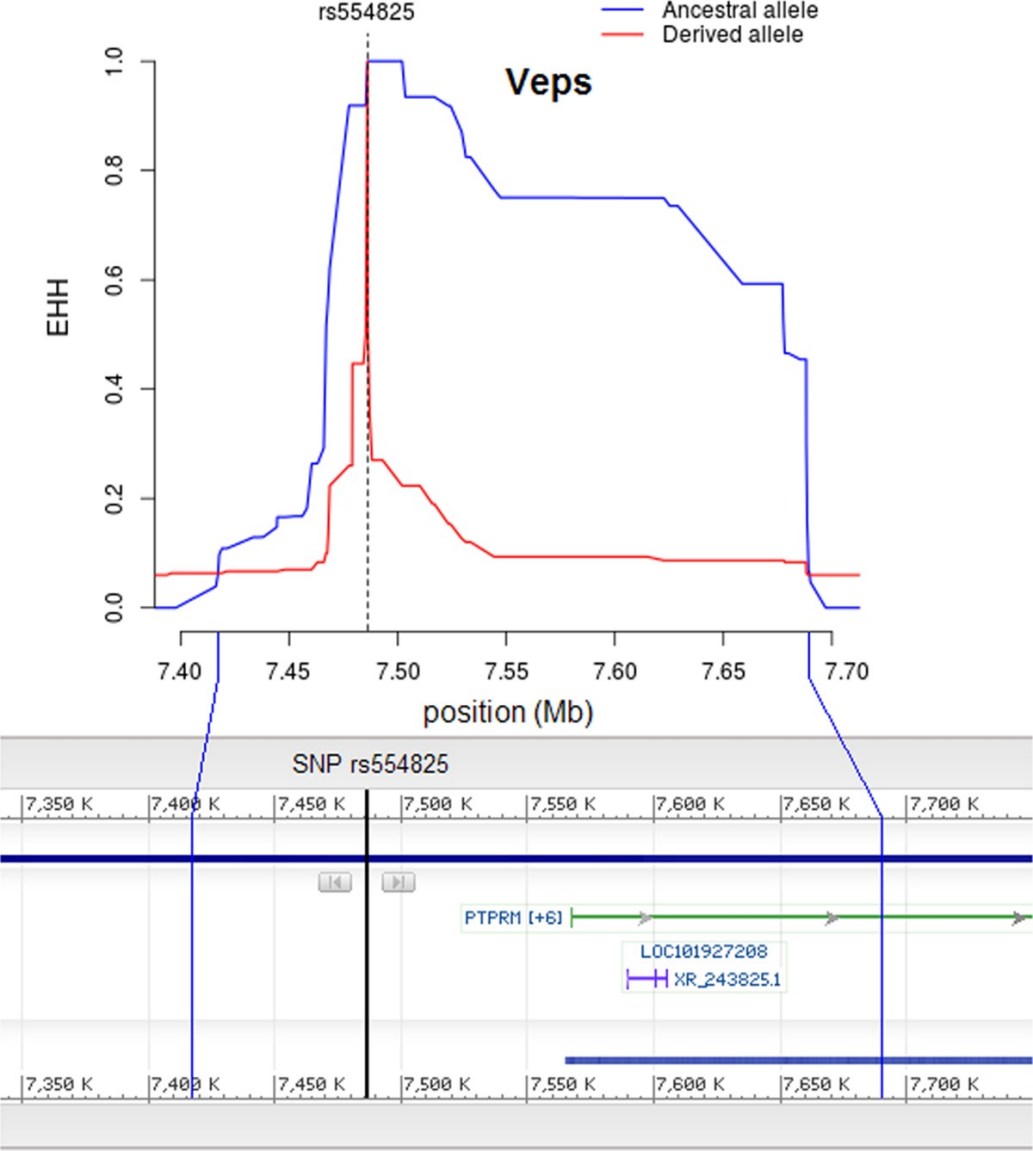
The decay of extended haplotype homozygosity in the region of SNP rs554825: Evidence from the Veps population. The bottom of the figure illustrates the location of the SNP in the corresponding part of chromosome 18, as at the NCBI variation viewer (GRCh37.p13), and the distances at which EHH for the ancestral allele drops to the threshold limit.

Genotypic datasets for eight populations from Northern Russia (Russians from the Archangelsk and Vologda regions, Izhemski Komi, Priluzski Komi, Veps, Khanty, Mansi and Nenets) were processed bioinformatically. According to the protocol of Voight et al. [27], raw integrated haplotype scores (iHS) for all SNPs that passed filters (call-rate for genotyping efficacy, minor allele frequency cutoff, and consistency with Hardy–Weinberg equilibrium) were binned by the frequency of derived alleles and normalized within each bin. The identification of candidate selected regions was performed using two approaches. The first method was based on picking out individual SNPs with prominent (*P* ≤ 1 × 10^−5^) iHS values in populations from Russia (Fig 2, S1 Fig). In total, 154 such SNPs were found, among which, nine had extreme iHS values in more than one population (S1 Table). These were the focus of our intense consideration because a SNP coincidence in different populations would increase the statistical significance of selection signals. Furthermore, many of the populations tested coexisted in the same geographical region and were coexposed to the same environment for a long time; hence, the same SNPs might often be selected. These nine SNPs from eight different loci are described in detail in Table 1. Two of them (rs554825 and rs10502389) were also replicated in European populations from the 1000 Genomes Project (CEU, FIN, TSI). Five of the nine SNPs (rs3738544, rs17014454, rs12774724, rs12769829, and rs958793) were located inside genes; thus, the corresponding candidate genes under selection were directly inferred from the SNP locations. Four other SNPs were within intergenic regions and the potential targets of positive selection associated with them could be outlined from the data on the decay of EHH around the SNPs of interest. For example, SNP rs554825 is located on chromosome 18 in the region between the *LRRC30* and *PTPRM* genes. Analysis of the decay of EHH for this SNP suggested that the *PTPRM* gene alone could be a target of selection (Fig 3). Many of the longest haplotypes carrying the ancestral allele of SNP rs554825 extended over the first 100 kb of the *PTPRM* gene; thus, it could affect the expression of this gene (Fig 3). SNP rs1387010, which is located on chromosome 4, seems to be also strongly associated with one candidate gene (*TOMM22P6*), while the remaining two SNPs–rs7695045 on chromosome 4 and rs10502389 on chromosome 18 –with four (*RN7SL101P*, *MIR4275*, *MESTP3*, and *LINC02364*) and five (*MTCL1*, *RPS4XP19*, *NDUFV2*, *ANKRD12*, *TWSG1*) genes, respectively (S2 Fig–S4 Fig).

**Table 1:** SNPs with significant (p ≤ 1 x 10^−5^) iHS scores found in populations tested.

SNP #rs ID	Chr	Position	Alleles	Selected allele	Functional consequence	Annotated genes	|iHS| score	iHS log P-value	Population**
rs3738544	1	236,914,576	C/T	C	Intron variant	*ACTN2*	4.5; 4.6	5.2; 5.4	Ru-Ust, Veps
rs17014454	3	24,277,049	C/T	T	Intron variant	*THRB*	4.4; 5.1	5.0; 6.4	Ru-Ust, Ru-Me
rs1387010	3	161,937,965	C/T	T	Intergenic	*TOMM22P6*	5.1; 4.9	6.0; 6.5	Veps, Ru-Me
rs7695045	4	28,850,524	A/G	G	Intergenic	*MESTP3*	5.1; 4.5	5.3; 6.4	Khanty, Mansi
rs12774724*	10	83,958,152	C/T	C	Intron variant	*NRG3*	5.6; 4.8	5.8; 7.7	Ru-Ust, Komi-Izh
rs12769829*	10	83,958,312	A/G	G	Intron variant	*NRG3*	5.6; 4.4	5.0; 7.7	Ru-Ust, Komi-Izh
rs958793	13	36,076,555	A/G	A	Intron variant	*NBEA*	5.0; 4.6	5.4; 6.3	Khanty, Nenets
rs554825	18	7,486,106	A/G	A	Intergenic	*PTPRM*	4.5; 5.2; 5.0; 4.9; 4.9	5.2; 6.6; 6.2; 6.0; 6.1	Veps, Ru-Me, TSI, CEU, FIN
rs10502389	18	8,978,472	A/G	A	Intergenic	*MTCL1*, *RPS4XP19*	4.9; 4.7; 4.9	5.9; 5.7; 5.9	Ru-Ust, Ru-Me, TSI

*The SNPs are very closely-spaced (located at 170 bp from each other) and thus can be considered as a single locus.

**Ru-Ust–Russians from Ustyuzhna, Ru-Me–Russians from Mezen, Komi-Izh–Izhemski Komi.

The relevance of the signals and candidate genes identified was additionally confirmed using the second approach, which was based on the analysis of SNPs in the 0.1% tail of the empirical distribution of iHS scores in non-overlapping 100 kb genomic windows across the populations tested. It was shown that all computational windows with the candidate genes described above had at least two top SNPs (|iHS| ≥ 3.17) in two or more populations tested. The highest number of top SNPs was observed in windows that included the *NRG3* and *NBEA* genes, as well as the region surrounding the *MSTP3* locus. The *NRG3* gene was the candidate region that was detected most frequently in the tested populations. *NRG3*-related SNPs with top iHS values were identified in all five populations from European Russia and two European samples from the 1000 Genomes Project (CEU and FIN). By contrast, *MSTP3*-related windows were only revealed in Siberian populations. The top SNPs that also occurred mainly among Siberians were located in the *NBEA* gene. Among other potential candidates for positive selection were the *TMEM229B* and *SEMA6A* genes, both of which contain SNPs with extreme iHS values in multiple populations (S1 Table).

### Genomic regions where signals of selection were revealed using the XP-EHH test

The dissection of the results of cross-population EHH (XP-EHH) calculations demonstrated the importance of several genomic regions where significant iHS values were detected previously. For example, multiple SNPs with significant (*P* ≤ 1 × 10^−5^) XP-EHH scores were identified in the regions of the *MESTP3*, *NRG3*, *NBEA*, and *PTPRM* genes (S2 Table). The *MESTP3* gene region had the highest number of SNPs with significant XP-EHH values. In total, 43 such SNPs were identified within this region. Interestingly, the significant SNPs in the *PTPRM* region were represented by two groups spaced 200 kb apart. The first was a distant group located around the rs554825 SNP described above and the second was localized directly in the *PTPRM* gene, in the middle of the first intron (chr18:7.678–7.689 kb). SNPs with significant XP-EHH values were also found in other chromosome regions (the complete list of SNPs with significant XP-EHH values in tested populations is presented in S2 Table (S2 Table).

Because the results of XP-EHH tests substantially depend on the choice of the reference population, we performed three sets of computations using the YRI, TSI, and CHB reference samples from the 1000 Genomes Project. The use of several reference populations may provide insights into the specificity of the distribution of selection signals observed[32]. The most illustrative patterns of occurrence of selected SNPs are shown in Table 2. For example, the *FGF2* and *BCAS3* genes were commonly subjected to selection in all non-African populations, while the *TMEM232*, *DUOX2*, *DUOXA1*, and *DUOXA2* genes were targets of selection in European populations only. *PTPRM* and *NRG3* could be additional “European” genes. The patterns of signals observed in Western Siberian samples were the most interesting, as they were independently shared between Europeans and East Asians (e.g., Chinese (CHB). *PDE11A* and *PPARD* seemed to be under selection in all populations with the exception of CHB. By contrast, the selected regions comprising the *PCSK9*, *KCNN3*, *SULT1C2*, *CYP26A1*, and *NIP7P1* genes were common among CHB and West Siberians. The signals of selection related to *WNT5B* were exclusively associated with West Siberian populations. Concomitantly, West Siberian Nenets were a stand-alone sample in their sharing of selection signals in the *EDAR* and *CDCP1* genes with the Chinese population.

**Table 2:** Candidate loci for positive selection identified using XP-EHH tests (p ≤ 1 x 10^−5^)*.

Chr	Posi-tion (Mb)	CEU	CHB	FIN	Komi-Izh	Khanty	Mansi	Ru-Me	Nenets	Komi-Ob	TSI	Ru-Ust	Veps	Genes and gene regions annotated
1	55.5		Y			Y, T	Y, T		Y, T					*PCSK9*
1	76.6		Y											*ST6GALNAC3*
1	154.8		Y			Y, T	Y		Y, T					*KCNN3*
1	189.5					Y	Y		Y					*RP11-373J16*.*2-RNA5SP73*
2	108.9		Y, T			Y, T	T		Y, T					*SULT1C2*, *RP11-443K8*.*1*, *SULT1C2P1*
2	109.6		Y						Y					*EDAR*, *EDAR-SH3RF3-AS1*
2	178.5	Y		Y	Y	Y	Y	Y	Y	Y	Y	Y	Y	*PDE11A*
3	45.1		Y, T						T					*CLEC3B-CDCP1*, *CDCP1*
4	28.7					Y, T	Y, T		Y, T					*RN7SL101P*, *MIR4275-MESTP3*, *MESTP3-LINC02364*
4	123.8	Y	Y	Y	Y	Y	Y	Y	Y	Y	Y	Y	Y	*FGF2*
5	109.6	Y		Y	Y			Y		Y	Y	Y, T	Y	*TMEM232*
6	35.4	Y		Y	Y	Y	Y	Y	Y	Y	Y	Y	Y	*PPARD*
6	136.1	Y		Y	Y	Y	Y	Y		Y	Y	Y	Y	*RP11-394G3*.*2*, *RP11-394G3*.*2-PDE7B*
10	83.9							C				Y, C		*NRG3*
10	94.8		Y, T			Y, T	Y, T		Y, T					*CYP26A1*, *CYP26A1-NIP7P1*
12	1.6					Y, T	Y, T		Y, T					*ERC1*, *ERC1-LINC00942*, *LINC00942*
12	1.6					T, C	T, C		T, C					*WNT5B*
12	89.2	Y		Y	Y			Y		Y	Y	Y	Y	*RNU1-117P-RP11-13A1*.*1*
13	36.1					Y, C	Y, C							*NBEA*
15	45.4	Y		Y	Y			Y		Y	Y	Y	Y	*DUOX2*, *DUOXA1*, *DUOXA2*
15	64.2		Y, T											*MIR422A-DAPK2*, *DAPK2*
17	28.5		Y			Y	Y		Y					*SLC6A4*
17	28.6		Y			Y	Y		Y					*TMIGD1*
17	59.2	Y	Y	Y	Y	Y	Y	Y	Y	Y	Y	Y	Y	*BCAS3*
18	7.5	Y		Y	Y			Y		Y		Y	Y	*LRRC30-PTPRM*
18	7.7	Y, C		Y, C	Y, C	Y		Y, C	Y	Y, C	Y, C	Y, C	Y, C	*PTPRM*

*Y, T, C–reference populations: YRI, TSI and CHB, respectively.

To determine whether any biological processes were targeted by selection in the populations studied, a gene ontology (GO) analysis was applied to the top loci identified. No categories were found to be significantly enriched for genes associated with iHS hits. A similar situation was with the genes selected based on XP-EHH tests, excepting Khanty population in which several GO categories were significant in the case of YRI reference (i.e., categories related to epithelial cell migration and proliferation).

### Genomic regions with signals of selection found by comparing allele frequencies from whole-genome sequences

#### Analysis of genome sequences in the region of loci depicted using EHH-based tests

In parallel with SNP microarray study, we explored public data on multiple sequenced genomes from North Eurasia and neighboring populations from the EGDP project[6]. First, we computationally searched the EGDP database of genomic sequence fragments representing eight loci from Table 1, which exhibited strong selection signals. Our goal was to find frequent alleles (MAF > 0.20) that were significantly overrepresented in Northern Eurasians (NEE and NWA groups) compared with five neighboring population groups conditionally designated as “Slavs”, “Europe”, “Altaians”, “Chukchi”, and “Tatars”; four distant regions from Eurasia (“Central Asia” [Kazakhstan, Tajikistan, Uzbekistan, Kirgizia], “Caucasus”, “Middle East”, and “India”); and a global human population sampled over several continents (so-called “Control” group). The groups are specified in column 4, while the individuals in columns 1–3 of the S3 Table. The populations are also described on Fig 1. SNPs with such requirements were found in the vicinity of five of these eight loci (Table 3). In three out of five cases, the over-representation of the alleles was statistically significant (P < 0.01). The identified SNPs are located inside introns or in intergenic regions. None of these SNPs are located in exons.

**Table 3:** SNPs of sequenced genomes within 9 loci described in Table 1*.

Locus	SNP #rs ID	Chr	Position	Derived allele frequency	SNP over-repre-sentence	P-value	Annotated genes
1	Not found	1	236,914,576				
2	Not found	3	24,277,049				
3	rs149915236 rs148419167 rs148368744 rs182084000 rs78230670 rs79428199 rs140812904 rs75151827 rs149123142 rs117166396 rs73172723 rs73172728	3	160,963,232 160,974,524 161,109,954 161,135,155 161,652,536 161,652,656 161,681,122 161,693,930 161,697,079 161,731,309 161,777,884 161,779,104	0.30 NWA	≥ x2.2	0.003	*NMD3*, *LOC107986150*, *TOMM22P6*
4	Not found	4	28,850,524				
5,6	rs1336274 rs61863039 rs72827309 rs10509451 rs61863041 rs61863047 rs61863048 rs17737264 rs61863049 rs61864201 rs11596426 rs72827335 rs75497737	10	84,028,864 84,029,031 84,029,228 84,032,154 84,034,357 84,038,763 84,039,605 84,042,161.84,042,276 84,070,366 84,071,127 84,074,280 84,074,407	0.30 NEE	≥x2.2	0.1	*NRG3*
7	rs147713651 rs117041926 rs147476568 rs111977790 rs112259913 rs190723450	13	35,159,886 35,162,526 35,182,715 35,456,591 35,481,662 35,550,828	0.21 NEE	≥x5.0	0.001	*NBEA*
8	rs141455074	18	8,441,576	0.28 NWA	≥x3.4	0.0001	*PTPRM* (3`-end)
9	rs67830720	18	9,493,156	0.34 NEE	≥x2.0	0.1	*RALBP1*

*Column 1 points to the rows from the Table 1 that describe SNPs from the same chromosomal regions. Column 5 shows the highest derived allele frequency of the identified SNPs observed in North West Asia (NWA) populations, located at the East of Ural Mountains, or North East Europe (NEE) populations (at the West of Ural Mountains). Column 6 presents the ratio of overabundance of the SNPs in North Eurasia populations in comparison to other populations under analysis. It shows how many times the frequency of SNPs in NEE or NWA is higher than in all other populations. Column 7 shows P-value–the estimated probability to find by chance the SNP overrepresented in North Eurasia populations with the observed ratio within the current loci (± 0.8 Mb from the location in Table 1). Column 8 presents genes that host the identified SNPs associated with signals of positive selection.

#### Exhaustive search of whole-genome sequences of positive selection

We also examined genotype data of whole-genome sequences of 402 individuals from the EGDP database and identified frequent SNP alleles (MAF > 0.25) in populations from Northern Eurasia that occurred at least three times more frequently there than they did in the remaining 10 populations, including five neighboring ones. For this computation, our thresholds became stricter than in previous paragraph to select the strongest sites throughout the whole genome (MAF was increased from 20 to 25%; while overrepresentation from x2.0 to x3.0 threshold). In addition, we examined whether the regions of these SNPs were among the signals of positive selection detected in any of the eight Northern European and Western Siberian populations examined using XP-EHH or iHS statistics (The *P*-value of iHS was set to < 1 × 10^−4^). This computation identified 24 SNPs that fulfilled all the requirements and were located in six different loci (Table 4). Some of identified SNPs from five loci were located within introns of the various genes listed in the table. None of these SNPs were located in exons. Among the genes carrying SNPs from Table 4, five are protein-coding and three are non-coding RNAs. The *P*-values for statistical significance of our genomic approach, which were calculated with Monte-Carlo simulations, were sufficiently low (< 1 × 10^−8^) and suggested that positive selection at all six loci from Table 4 is not a random fluctuation of allele frequencies. The strongest selection signal was observed for six SNPs on chromosome 11 shown at the bottom of Table 4. The alternative alleles of these six SNPs have frequencies in the Siberian populations (NWA) between 26.5% and 30.4%, which is at least nine times higher than the frequencies observed in the remaining 10 population groups under study. These six SNPs are spread over a 211 kb chromosomal region and are located inside introns of the *SLC37A2* and *PKNOX2* genes. Several other genes with unknown functions, including ncRNAs, are also present within this locus.

**Table 4:** Six loci with the strongest signals for positive selection in North Eurasia populations identified by whole-genome allele frequency analysis*.

SNP #rs ID	Chr	Position	Haplo-type size (kb)	Derived allele frequency	SNP over-repre-sentance	P-value	Annotated genes
rs77191500 rs75647011 rs77338913 rs78178069	3	54,081,807 54,082,785 54,083,724 54,084,458	4	0.34 NWA	≥x4.4	<10^−8^	*CABYRP1* (pseudogene)
rs17198295 rs41345846 rs115064616 rs4678429 rs116387571 rs76578737 rs74641205	3	135,856,318 135,956,639 135,966,726 135,993,276 136,339,949 136,350,891 136,374,804	500	0.30 NEE	≥x4.3	<10^−7^	*PPP2R3A*, *PCCB*, *STAG1*
rs77693347	3	143,571,375		0.26 NWA	≥x3.2	<10^−8^	*SLC9A9* (promoter)
rs62469388 rs62471762 rs62471765 rs62471766 rs62471768	7	89,038,148 89,168,136 89,195,490 89,228,812 89,253,624	210	0.27 NEE	≥x5.3	<10^−8^	*lincRNA XR_001745266* (promoter)
rs117000964	11	40,094,935		0.26 NWA	≥x3.2	<10^−8^	*LOC105376637*
rs118138358 rs75705739 rs36015256 rs148184827 rs117952463 rs74566282	11	124,948,795 124,954,830 125,074,999 125,085,473 125,103,343 125,159,749	210	0.30 NWA	≥x9.0	<10^−8^	*SLC37A2*, *PKNOX2*

*Column 4 –presents size of the haplotype formed by the SNPs (the distance between the first and the last SNP from this locus). Column 5 –the highest derived allele frequency of the SNPs observed in NWA or NEE populations. Specific geographic locations and list of individuals with these alleles are listed in the S3 Table. Column 6—ratio of overabundance of these SNPs in North Eurasian populations in comparison to other populations under analysis. Column 7 shows P-value–the estimated probability to find by chance the SNP overrepresented in North Eurasia populations with the observed ratio. Column 8 presents genes that host the detected SNPs associated with positive selection.

Previous analyses have not revealed a single SNP located inside protein-coding regions. To clarify this issue, we computationally processed 402 EGDP genomes and found all SNPs with alternative alleles that change protein sequences. We further filtered these SNPs to meet the following two requirements: 1) MAF frequencies > 0.20 in Northern Eurasia, and 2) over-representation in Northern Eurasia (at least 1.8 times compared with all other 10 populations under analysis). We lowered the thresholds in this analysis because the original ones did not reveal a sole SNP. With new threshold conditions we found seven such SNPs, all of which represented missense mutations, which are listed in Table 5. These are the top candidates of mutations that change protein structure and may be under positive selection in Northern Eurasia.

**Table 5:** List of most prominent missense mutations that are overrepresented in North Eurasia.

SNP #rs ID	Chr	Position	Mutation Ref -> Alt	Gene	Over-repre-sentance	Population
rs12039961	1	203,276,546	G->A	*BTG2*	≥x1.8	NWA
rs147417448	7	47,894,518	T->A	*PKD1L1*	≥x2.2	NWA
rs17132289	7	48,428,715	A->T	*ABCA13*	≥x1.9	NWA
rs78225095	7	99,725,146	C->G	*MBLAC1*	≥x2.3	NWA+NEE
rs78279132	7	100,137,085	G->C	*AGFG2*	≥x2.5	NWA
rs143390591	15	75,980,291	T->C	*CSPG4*	≥x1.8	NWA
rs116676388	19	19,613,284	G->A	*GATAD2A*	≥x2.6	NWA
rs16986050	19	55,401,170	A->G	*FCAR*	≥x1.9	NEE

Column 1 shows SNPs inside coding regions that have signals of positive selection revealed by allele frequency analysis. Column 2 presents the number of the chromosome containing the described SNP. Column 3 shows position of these SNPs on chromosomes. Column 4 –mutations that change coding sequences. Column 5 presents genes that host the detected SNPs associated with positive selection. Column 6—ratio of overabundance of these SNPs in North Eurasian populations in comparison to other populations under analysis. Column 7 –North Eurasian populations where these derived alleles are overrepresented.

## Discussion

### Microarray data analysis

In the current study, we performed genome-wide scans of EHH in populations from Western Siberia and Northern European Russia. Via comparisons of the two alleles of particular SNPs, the ratios of integrated EHH (iHS) provide valuable information on selection strength at each locus, and the subsequent analysis of corresponding EHH profiles allows outlying the regions and genes under selection [27]. Eight such chromosomal regions were identified, four of which (i.e., the regions associated with the locations of the *MESTP3*, *NRG3*, *NBEA*, and *PTPRM* genes) were also revealed in cross-population testing of EHH (XP-EHH test).

The *MESTP3* and *NBEA* loci were shown to be under selective pressure for the first time. This situation seemed to be the result of the absence of appropriate populations in the previous studies (i.e., Western Siberian populations (Khanty, Mansi, and Nenets). *NBEA* encodes the brain-enriched scaffolding protein neurobeachin, which is involved in membrane trafficking and synaptic functioning [33]. It has been identified as a candidate gene for neurodevelopmental diseases, including autism [34]. All selection signals associated with *NBEA* were inside the gene and intronic. The *MESTP3* genomic region provided a more interesting case. *MESTP3* is a non-coding gene (pseudogene) [35]. Moreover, none of the other genes in the region, including *RN7SL101P*, *MIR4275*, and *LINC02364*, encoded proteins. Their products are affiliated with long non-coding RNA or microRNA classes. Pseudogenes themselves can also be a source of small interfering RNAs, another RNA class resembling microRNAs [36]. All of these RNA types play important roles in the regulation of diverse cellular processes, ranging from gene transcription to chromosome remodeling and intracellular trafficking [37–39]. One can propose that cooccurrence of these different RNA genes within a certain signature of selection is not random. They can result in a functional complex with a broad regulatory capacity that would influence local population adaptation, particularly Western Siberian populations.

The *NRG3* and *PTPRM* genes have been listed as potential targets of natural selection [15,40]. Neuregulin 3 is a key component of the NRG3–ERBB4 pathway, which is involved in the development of several tissues, with strongest effects on the differentiation of the neural system [41]. NRG3 has been implicated as a susceptibility gene for schizophrenia and schizoaffective disorders [41]. *PTPRM* encodes a protein tyrosine phosphatase with an extracellular region that functions as a receptor involved in mediating cell–cell interactions and adhesion. *PTPRM* expression has been shown to be negatively correlated with oncogenic cell growth and cancer prognosis [42,43]. In agreement with the work of Pickrell et al. [40], both iHS and XP-EHH extreme values in *NRG3* and *PTPRM* were mainly detected in European populations. The signals associated with *NRG3* were all located inside the gene. At the same time, our results showed that the signals related to *PTPRM* were represented by two distinct groups, one associated with an intergenic region located at 80 kb from the start of the gene, and the other located in the middle of the first intron. Because *PTPRM* is a large differentially expressed and alternatively spliced gene [44], one can propose that such structure of patterns of variability plays a role in the regulation of the expression of the longest forms of *PTPRM*.

In the strategy used by us for searching for selection signals, we paid particular attention to the significant SNPs that occurred in more than one population. Such SNPs were found in populations from the same geographical regions (e.g., North Eastern Europe and Western Siberia). Some of those detected in populations from the European part of Russia were also found in European populations from the 1000 Genomes Project. This was unsurprising in the context of the patterns of population structure commonly shared among Europeans [45,46]. Additional support for the sharing of selection signals between populations with similar location and demography were obtained in XP-EHH tests. In contrast to the common approach, we simultaneously applied three different reference populations—YRI, TSI, and CHB. By doing this, we expected to outline the geography of the patterns of selection signals in the regions [47]. Western Siberia, exemplified by Khanty, Mansi, and Nenets, was of particular interest. We found that many of the signals identified were independently shared with both Europeans and East Asians, which might be attributed to the complex population history of Khanty, Mansi, and Nenets making them related to both Europeans and Asians [10]. It has been recently shown that Khanty, Mansi, and Nenets are descendants of ancient Northern Eurasians (ANE) and Eastern Siberians[10]. ANE people are believed to be basal to modern-day Western Eurasians, particularly Europeans, with no close affinity to East Asians [8]. At the same time, Eastern Siberian people are a sub-lineage of East Asian communities that diverged about 10,000 years ago[10]; thus, being related to Eastern Siberians, Western Siberians are also related to East Asians (e.g., Chinese). In that context, the observation that the signals located in some genes were shared just between Nenets and CHB was of special interest. One of those genes was *EDAR*. It is required for the development of different ectodermal derivatives, including hair, teeth and sweet glands, and is a well-known target of selection in Asian (East Asian) populations [20,48]. Another such common gene in Nenets and CHB was *CDCP1*, which was established to be involved in cell adhesion. Summarizing the above, one can propose that stand-alone position of Nenets among Western Siberians is attributed to the greater proportion of East Asian ancestry in Nenets, which they additionally obtained from Eastern Siberians, after the split from Khanty and Mansi [10].

The group identified and the regional population specificity in the distribution of selected signals agree with the results of previous studies [24,47,49], which showed that the signals shared among populations followed the patterns of the population structures observed. Taken together, these results suggest that local adaptation is tightly constrained by ancestral relationships between populations.

### Whole-genome sequence analysis

The results of studies that searched for signals of local adaptations are more reliable if they are supported by different algorithms [50]. To verify the findings obtained using SNP microarrays, two subsets of whole-genome sequences from the EGDP dataset were created and examined for loci that occurred more frequently in populations from Northern Eurasia than in other populations across the world. We observed a good correspondence of the revealed selection signals between SNP microarray statistical analyses and examinations of whole-genome sequences from Northern Eurasia based on allele frequencies in the EGDP dataset. In fact, the first putative selection site for the latter approach presented on Table 4 (chromosome 3, position 54 Mb) also gave one of the top iHS hits (10^−8^) in the FIN population. The whole-genome analysis also detected the high occurrence of the alternative alleles of SNPs under selection in this locus (rs77191500, rs75647011, rs77338913, and rs78178069) in three sequenced Finnish and three Saami people from the EGDP database. One Finnish person (GS0000018756) was homozygous for these four alternative alleles while two Saami (GS0000035024 and GS0000035026) were heterozygous. A better correspondence between the SNP-microarray and genome sequence analysis approaches was observed after lowering the thresholds for MAF and/or allele over-representation level. However, lowering the thresholds may compromise the statistical significance of the results; thus, we did not follow this path. Whole-genome sequencing finds all genetic variations in individuals. Hence, it should allow finding the SNPs that are prime targets for selection. Similar to the SNPs from the microarray part, all 57 SNPs with the strongest signals for selection from Tables 3 and 4, which were found by whole-genome sequence examination, are either in the middle of very large introns or inside intergenic regions. Such priority of location of SNPs under selection corresponds well with the data of other publications, which demonstrated that selection signals are pervasive in non-coding regions [51]. This suggests that selection preferably operates through modulatory and regulatory mechanisms, instead of changes in protein structure.

The strongest selection signal detected by whole-genome sequencing came from chromosome 11 at position 124 Mb. The six SNPs with selection signals from this locus spanned over 211 kb and were located inside the introns of two genes: *SLC37A2* and *PKNOX2*. PKNOX2 belongs to the highly conservative TALE (3-amino acid loop extension) class of homeodomain proteins that play fundamental roles in cell proliferation, differentiation, and death. Genome-wide associated studies revealed that *PKNOX2* is associated with alcohol addiction in mice and humans [52–56]. Although its role in alcohol dependence remains largely unclear, our finding is of special interest in the context of the well-known alcohol addiction that occurs widely in indigenous populations of Northern Eurasia. The second gene, *SLC37A2*, is a member of endoplasmic reticulum-associated sugar-phosphate/phosphate (P(i)) exchangers. A recent study found that *SLC37A2* contains a conserved vitamin D receptor (VDR)-binding site [57]. This study also showed that the expression of *SLC37A2* in human peripheral blood mononuclear cells depends on the circulating form of vitamin D_3_. Deficit of vitamin D is often observed in northern populations, where exposure to sunlight is limited for many months. Hypothetically, mutations in the VDR-controlled *SLC37A2* gene may help northern populations adjust to vitamin D levels. At the same time, the same mutations could have effects on alcohol tolerance in these populations through the *PKNOX2* gene, located on the opposite strand of DNA in the same locus. Further studies of the expression of these two genes in Northern Eurasian populations are required to shed light on this problem.

We also found seven SNPs in protein-coding regions whose derived alleles occurred substantially higher in Northern Eurasia. These SNPs might be potential targets of selection. The effects of two of them on risk of some medical problems had been demonstrated. For example, the SNP rs147417448 was significantly (P-value = 10^−35^) associated with metabolic syndrome in Japanese populations [58]. Another SNP (rs16986050) was associated with one of the forms of autoimmune vasculitides–granulomatosis and polyangiitis (GPA). Change of A allele to G at codon 248 results in lower susceptibility to GPA [59]. Interestingly, the prevalence of GPA is more frequent among populations of Northern of Europe and America while in the south of these continents and in Asia other forms of autoimmune vasculitides are more common [60–62].

In conclusion, our study provided novel data toward establishing a genomic map of human local adaptations in Northern Eurasia, particularly in Russia, one of the largest and most multifaceted countries of the world, regarding both environment and ethnicity. In total, it outnumbered all studies performed before and demonstrated the fruitfulness of inclusion of new populations (populations from new geographic regions) for uncovering the molecular ways used for adaptation. Many of the described adaptation SNPs seemed to result from the involvement of non-coding RNAs. Several loci affiliated with different classes of non-coding RNAs were found to be under selection, particularly in the region of chromosome 4 (chr4:28.7–28.9 Mb), which contained the largest amount of significant selection signals. Cross-population testing suggested a role for this chromosomal region in the local adaptation of Western Siberians. It was also interesting to find among the top targets of positive selection the genes with known roles in the functioning and development of the nervous system (e.g., *NRG3* and *NBEA*) that confirmed the proposed great role of such genes in local adaptations of humans after they left Africa. Generally, the distribution of both these and many other selection signals demonstrated geographic and group (regional) specificities, suggesting the contribution of population structure and ancestry to the adaptation. Information on regional specificity in the distribution of the signals combined with the results of epidemiologic and animal model studies can be useful for clarifying the functions of particular genes (e.g., *SLC37A2* and *PKNOX2*).

## Materials and methods

### Ethics statement

The study was approved by the Ethics Committee of Institute of Molecular Genetics of the Russian Academy of Sciences, Moscow, Russia (statement no. 1 from 14 March, 2016).

### Study samples and genotyping

DNA samples of Russians from the Ustyuzhna district of the Vologda region (N = 46) and of Nenets from the Yamal peninsula (Yamalo-Nenets Autonomous Okrug) (N = 48) were genotyped for the first time using Illumina OmniExpress-24 arrays, according to the manufacturer’s protocol. The genotype data has been uploaded to the European Genome-phenome Archive and is accessible with EGA accession number EGAS00001003955. DNA was isolated from peripheral leukocytes according to standard techniques using proteinase K treatment and phenol–chloroform extraction [63]. Blood samples were collected in EDTA-coated vacutainers after interviewing and obtaining informed consent from each individual. Inclusion in the study required that all individuals belong to the native ethnic group of the region studied (i.e., they belonged to at least the third generation living in a particular geographic region) and were unrelated. Genotype data of the other populations analyzed–Russians from the Archangelsk region (Mezen district), Izhemski Komi, Priluzski Komi, Veps, Khanty, and Mansi which were genotyped with Illumina OmniExpress arrays–were obtained from two previous studies [10,46]. All samples were subjected to the same quality-control procedure using Plink software (v1.90b5.2) [64]; samples with a genotyping call rate < 0.95 were excluded. Although all volunteers were enrolled in the study based on questionnaires, the genotype datasets were additionally checked for the presence of cryptic relatedness: from the detected relative pairs (PI_HAT > 0.2), only one sample was chosen (e.g., an individual with a higher call rate of genotyping). Only autosomal genotypes were subjected to further analysis. The raw data sets were cleaned to remove duplicate entries and SNPs with zero genomic coordinates. The human genome assembly GRCh37 was used. To obtain some well-described reference populations, the same SNPs of unrelated individuals from TSI, FIN, CEU, CHB, and YRI populations were selected from phase 3 of the 1000 Genomes Project [65,66]. The relatives were identified according to the enclosed pedigree file.

As we were interested in cross-population statistics (e.g., XP-EHH), we subjected to further analysis the common SNPs for the two data sets that were genotyped by us and taken from the 1000 Genomes Project. The genomic coordinates of our samples were unified to those of 1000 Genomes. Thus, 672559 SNPs were common. The final sample sizes were as follow: Veps (N = 72), Izhemski Komi (N = 74), Priluzski Komi (N = 69), Russians from the Archangelsk region (N = 93), Russians from the Vologda region (N = 45), Khanty (N = 42), Mansi (N = 37), and Nenets (N = 41).

### Computation of iHS and XP-EHH scores

iHS and XP-EHH tests require haplotypes for computations [27,28]. The 1000 Genomes data sets were already phased. Therefore, we needed to phase only the populations that were genotyped by us, which was achieved using SHAPEIT software (v2.r837) [67] and applying the 1000 Genomes European subset as the reference panel. Before the phasing was performed, the SNPs were additionally filtered to exclude those that met the following requirements: minor allele frequency (MAF) < 0.01, significance threshold of the Hardy–Weinberg equilibrium test < 1 × 10^−5^, and missing rate per SNP > 0.05. All data manipulations at this stage were also made using the Plink tool.

The iHS and XP-EHH tests were performed using the REHH2.0 package [68]. For each SNP, the ancestral state was identified from chimpanzee genomic data downloaded from Ensembl (release 91) [69]. SHAPEIT output files, as well as VCF files from the 1000 Genomes Project, were converted into the input files for the REHH2.0 package using custom scripts in R[70].

The default arguments of the REHH package were used to estimate EHH, iHS, and XP-EHH values; 200 kb regions located near the centromere and chromosomal ends were excluded from the analysis because of possible artifacts caused by the marginal effects of these tests. In the case of XP-EHH computations, eight of our populations and four populations from the 1000 Genomes Project (CEU, FIN, TSI, and CHB) were tested in pairs with YRI, CHB, and TSI. Only positive XP-EHH scores corresponding to the suggested signals of selection in non-reference populations were subjected to further analysis. In the case of iHS data, the absolute values of the scores were used. As iHS and XP-EHH are constructed to have an approximately standard Gaussian distribution the *P* value cutoffs can be obtained from a Gaussian fit to the resulting data [68,71]. To convert the obtained iHS and XP-EHH scores the Gaussian cumulative distribution function implemented into REHH2.0 was applied. SNPs with *P* values ≤ 1 × 10^−5^ were considered as the signals of selection. Such SNPs were further annotated using the snpEff program (v.4.3p) [72]. Additionally, genes or genomics regions subjected to selective pressure were searched in 100 kb non-overlapping genomic windows contained SNPs found in the 0.1% tail of the empirical distribution of the test statistics [27,73,74]. Windows with two or more such SNPs were filtered and SNPs from them were annotated. The signals in the region of location of the major histocompatibility complex on chromosome 6 were not specially considered because of the complex structure of the region (e.g., high levels of variation, extensive linkage disequilibrium, and the highest density of genes in the human genome) [75,76].

The lists of genes that resulted from the annotation were checked for over-representation in terms of Gene Ontology using the WebGestaltR package, which represents an R interface of the WEB-based GEne SeT AnaLysis Toolkit [77].

The exploratory analyses of the results obtained at each stage of the study, as well as the visualization of the data sets obtained, were made with custom scripts in R.

### Computational processing of the EGDP whole-genome sequencing database

SNPs of whole-genome sequences of 402 individuals from the EGDP database were downloaded in a multi-sample VCF format from the site [29]. To obtain appropriate sample sizes the individuals from this database were pooled into 12 population groups based on their linguistic and ethnic similarity, as well as proximal geographical locations. These groups are specified in the S1 File. Eleven population groups were from different regions of Eurasia, whereas the last group, the so-called “control” group, comprised individuals from other continents, including Africa and South America. Two groups that intersected with the populations under study that had been tested with SNP microarrays were named “NEE: North-East-Europe” and “NWA: North-West-Asia”. NEE included 28 individuals from the Veps, Komi, Karelians, Ingrians, Estonians, Northern Russians, Finnish, and Saami populations, while NWA included 51 people from the Evenks, Evens, Forest Nenets, Khanty, Mansi, Kets, Nganasans, Selkups, Shorts, Tundra Nenets, and Yakuts populations. The table *EGDPindividuals*.*txt* describing all individuals and the corresponding population groups are presented in S1 File. Five groups (“Slavs”, “Europe”, “Altaians”, “Tatars”, and “Chukchi”) were in geographical contact with our two tested samples, NEE and NWA, and some gene flow occurred historically between them [5].

To study the SNP allele frequencies within each group, we wrote the Perl script AlleleFreqEGDP.pl, which is also available for public use from S1 File and has a description inside it. This program finds over-represented alleles in a specified tested group in which the frequencies are several times higher than those in the remaining groups. As input parameters, this program takes two thresholds: 1) the MAF value in the tested group and 2) the minimal times of allele over-representation (e.g., 3 times; 3×) of an allele in the tested group compared with the other groups. When these two thresholds were too stringent resulting in no hits, they were relaxed. Particularly, for data in Table 5 we used allele over-representation >1.8 times.

## Supporting information

S1 File Details of computational processing of the EGDP whole-genome sequencing database. (GZ)

S1 Fig Genome-wide (autosomes 1–22) distribution of p-values for iHS scores in the populations (from top to bottom) Khanty, Mansi, Nenets, Izhemski Komi, and Priluzski Komi. Horizontal red lines indicate P-value threshold applied (P ≤ 1 x 10^−5^). Loci of interest are pointed with arrows.
(TIF)

S2 Fig The decay of extended haplotype homozygosity in the region of SNP rs1387010: evidence from the Russian population from the Mezen district. The bottom of the figure illustrates the location of the SNP in the corresponding part of chromosome 3, as at the NCBI variation viewer (GRCh37.p13), and the distances at which EHH for the ancestral allele drops to the threshold limit.
(TIF)

S3 Fig The decay of extended haplotype homozygosity in the region of SNP rs7695045: evidence from the Mansi population. The bottom of the figure illustrates the location of the SNP in the corresponding part of chromosome 4, as at the NCBI variation viewer (GRCh37.p13), and the distances at which EHH for the ancestral allele drops to the threshold limit. Note: *LINC02364* is absent in GRCh37 (it appears in GRCh38).
(TIF)

S4 Fig The decay of extended haplotype homozygosity in the region of SNP rs10502389: evidence from the Russian population from the Mezen district. The bottom of the figure illustrates the location of the SNP in the corresponding part of chromosome 18, as at the NCBI variation viewer (GRCh37.p13), and the distances at which EHH for the ancestral allele drops to the threshold limit.
(TIF)

S1 Table SNPs with significant iHS values identified in populations tested. (XLSX)

S2 Table SNPs with significant XP-EHH values identified in populations tested. (XLSX)

S3 Table Loci with the strongest signals for positive selection in North Eurasia populations identified by whole-genome allele frequency analysis. File includes complete list of individuals whose genome sequences have been analyzed. The table contains the EGDP identifiers of each person (in the first column), the population name (in the second column), the country of origin (in the third column), and the name of the corresponding population group (one of our 12 groups) (in the fourth column). Columns representing SNPs from Tables 3 and 4 are highlighted in yellow or red. The alleles in these columns are presented in a standard VCF format: 0|0 means the person is homozygous with reference allele, 1|1 means that the person is homozygous with alternative allele, while 0|1 or 1|0 means that the person is heterozygous for this SNP.
(XLSX)
