# Supplementary figures and images for: Genomic landscape of the signals of positive natural selection in populations of Northern Eurasia: A view from Northern Russia
Source: PLoS One. 2020 Feb 5;15(2):e0228778. doi: 10.1371/journal.pone.0228778 (PMC7001972; doi:10.1371/journal.pone.0228778)

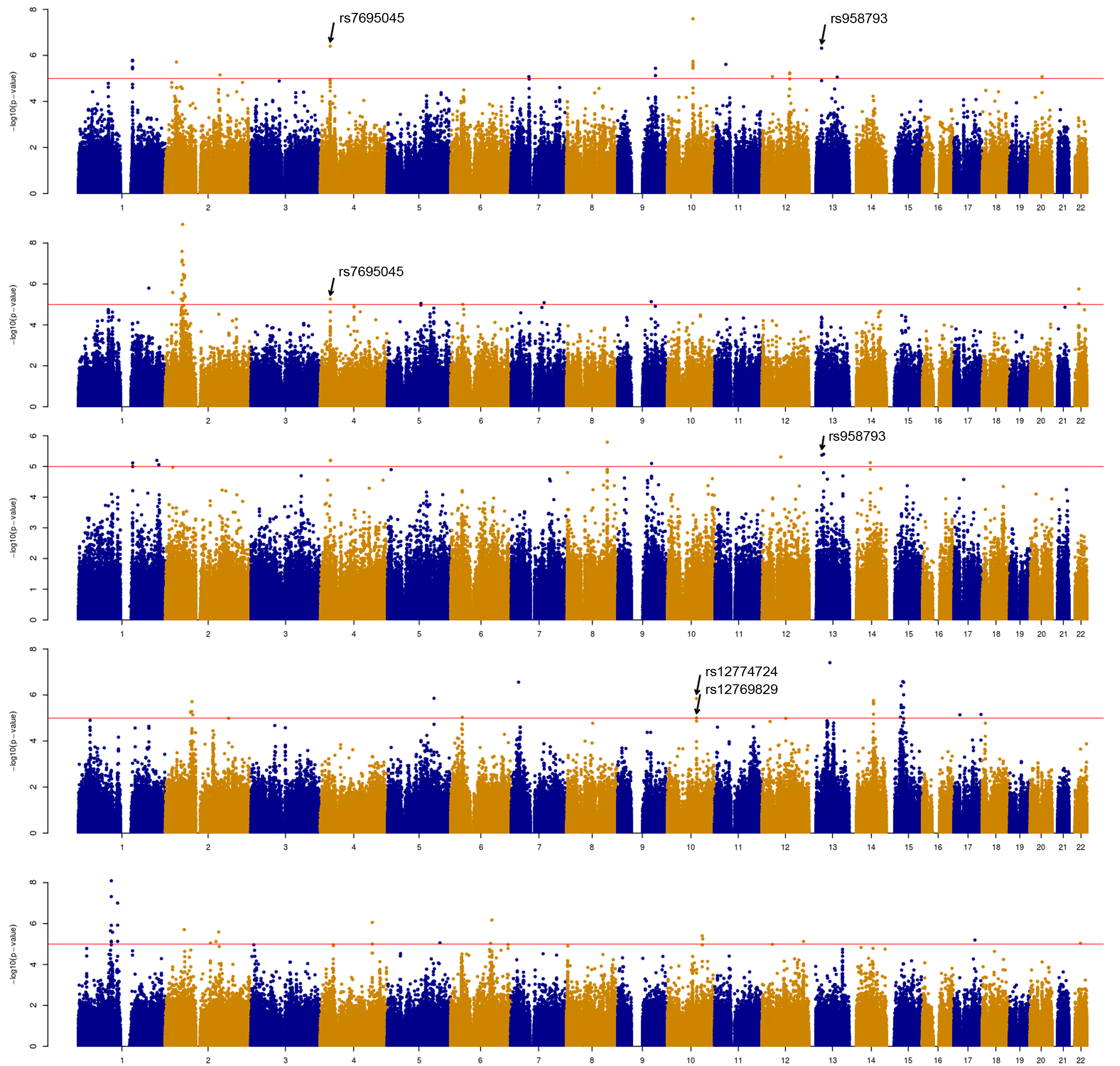

Supplement: S1 Fig — Horizontal red lines indicate P-value threshold applied (P ≤ 1 x 10^−5^). Loci of interest are pointed with arrows. (TIF) [file pone.0228778.s002.tif]

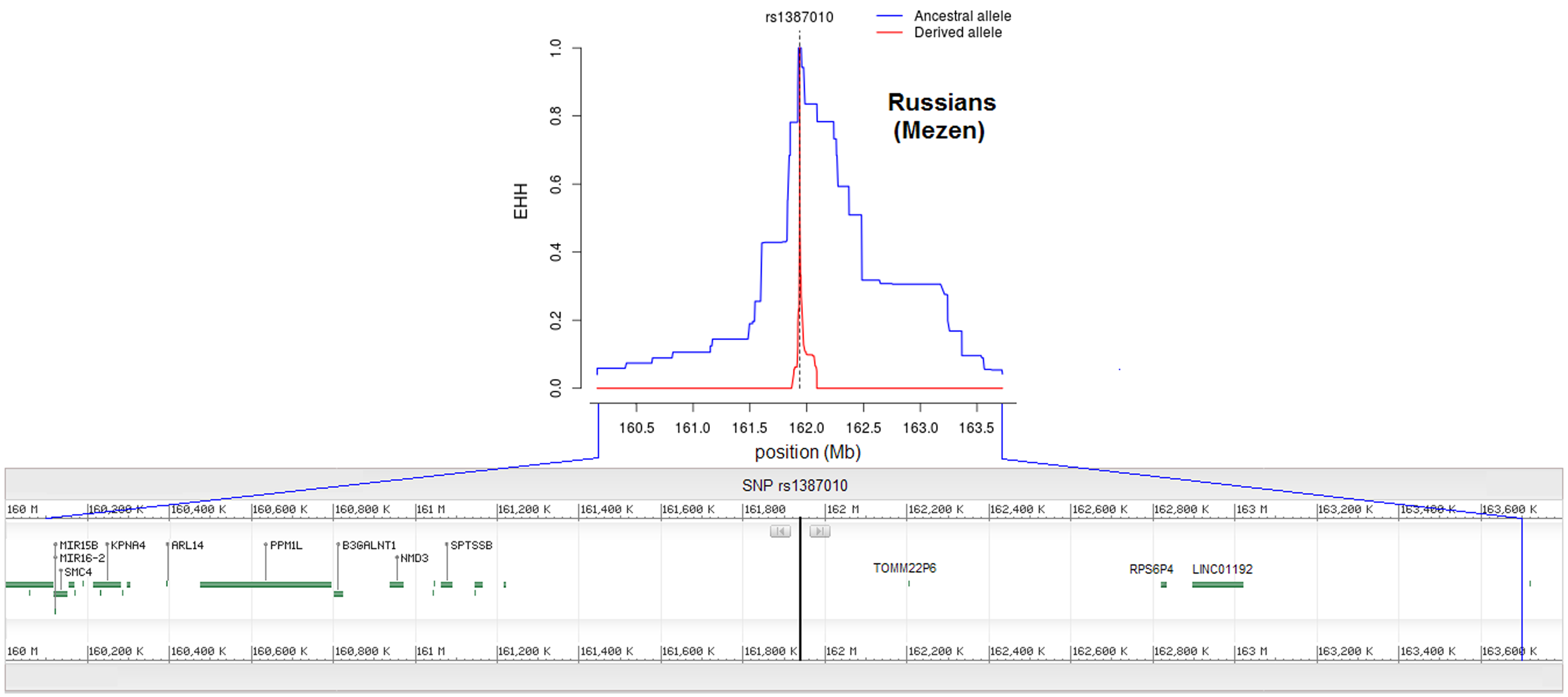

Supplement: S2 Fig — The bottom of the figure illustrates the location of the SNP in the corresponding part of chromosome 3, as at the NCBI variation viewer (GRCh37.p13), and the distances at which EHH for the ancestral allele drops to the threshold limit. (TIF) [file pone.0228778.s003.tif]

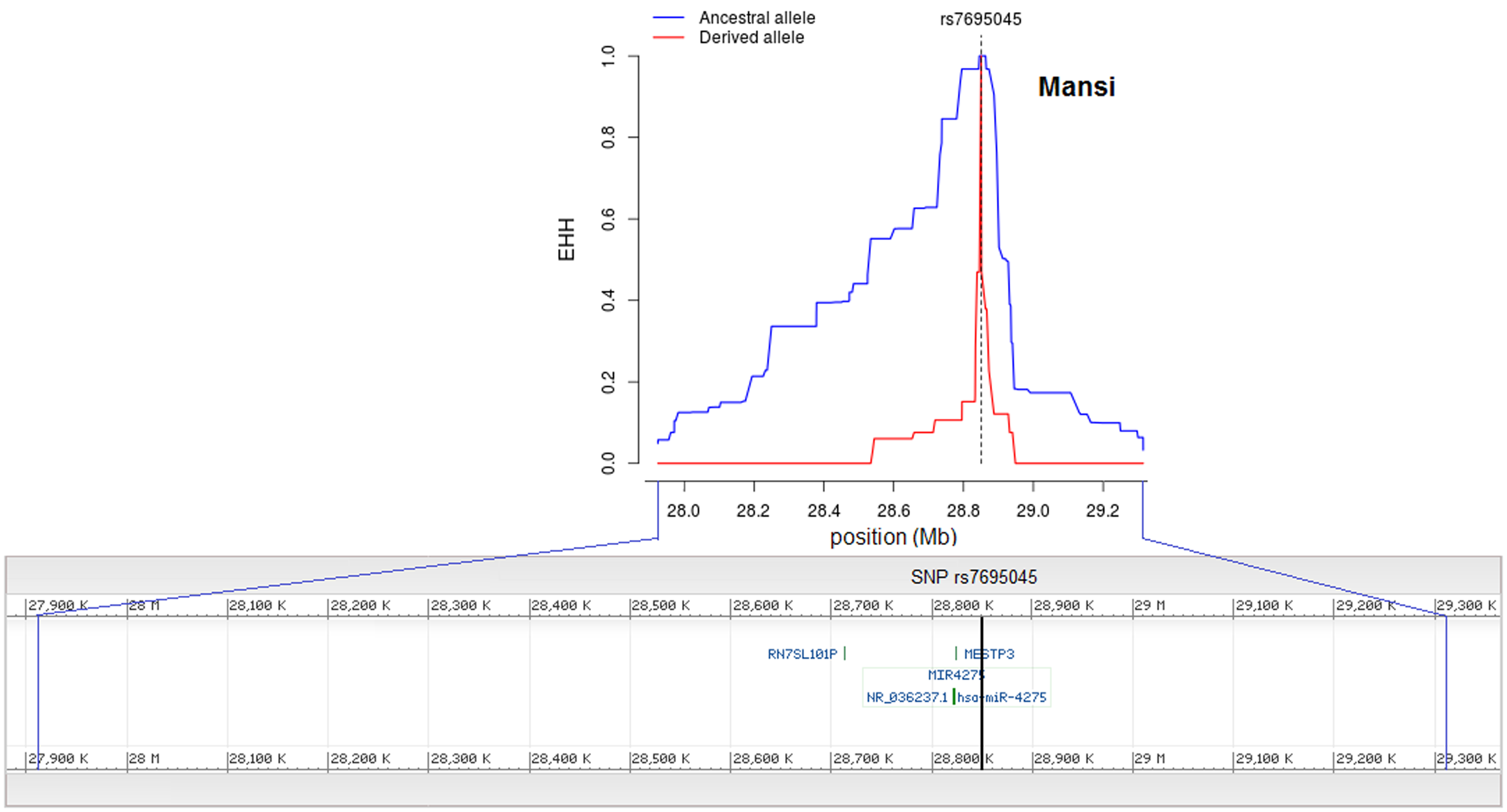

Supplement: S3 Fig — The bottom of the figure illustrates the location of the SNP in the corresponding part of chromosome 4, as at the NCBI variation viewer (GRCh37.p13), and the distances at which EHH for the ancestral allele drops to the threshold limit. Note: LINC02364 is absent in GRCh37 (it appears in GRCh38). (TIF) [file pone.0228778.s004.tif]

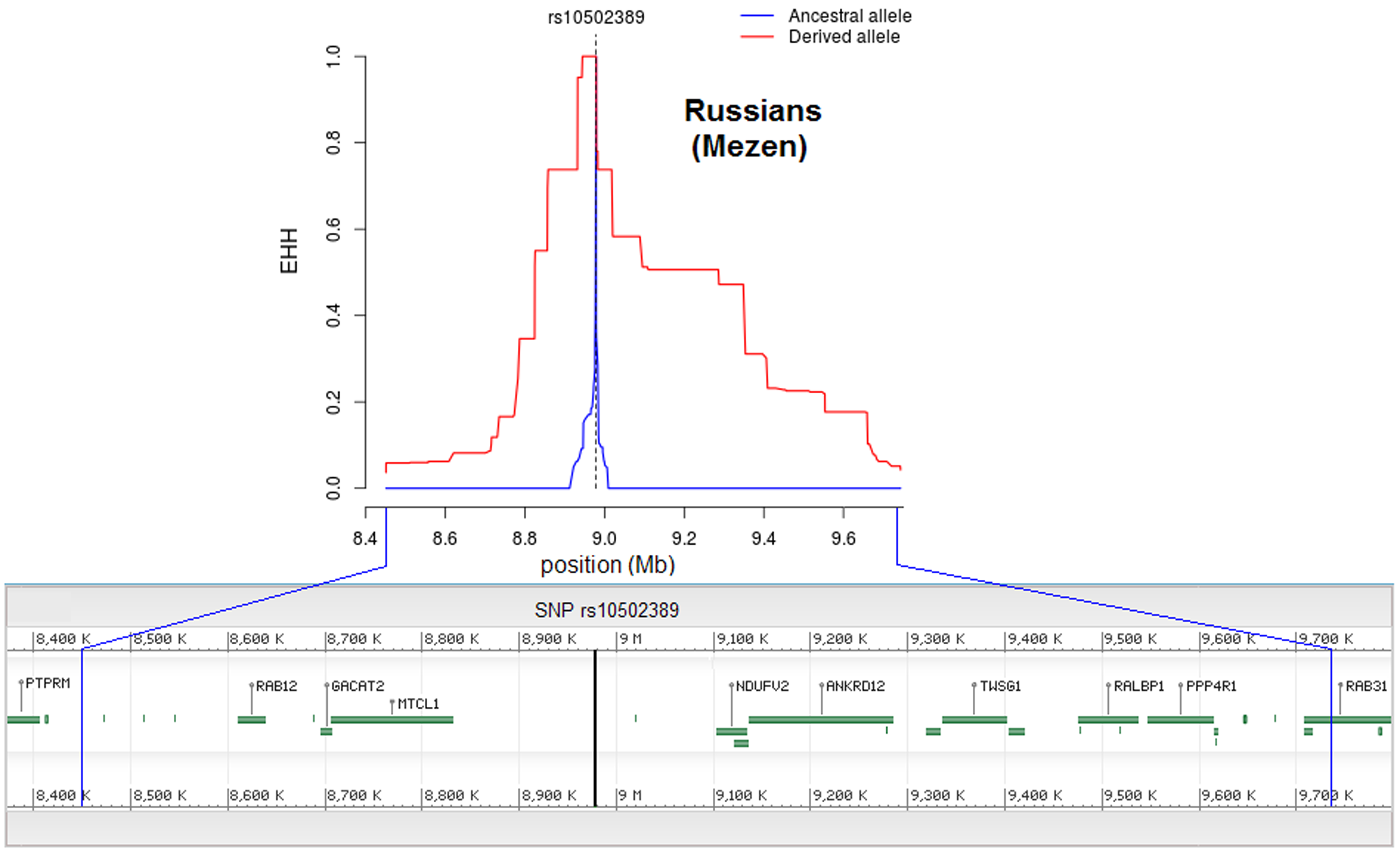

Supplement: S4 Fig — The bottom of the figure illustrates the location of the SNP in the corresponding part of chromosome 18, as at the NCBI variation viewer (GRCh37.p13), and the distances at which EHH for the ancestral allele drops to the threshold limit. (TIF) [file pone.0228778.s005.tif]
